# Seat belt and mobile phone use among vehicle drivers in the city of Doha, Qatar: an observational study

**DOI:** 10.1186/s12889-015-2283-3

**Published:** 2015-09-22

**Authors:** Ziyad R. Mahfoud, Sohaila Cheema, Hekmat Alrouh, Mohammed Hamad Al-Thani, Al Anoud Mohammed Al-Thani, Ravinder Mamtani

**Affiliations:** Division of Global and Public Health, Weill Cornell Medical College in Qatar. Qatar Foundation, Education City, P.O. Box: 24144, Doha, Qatar; Department of Healthcare Policy and Research, Weill Cornell Medical College, 402 East 67th Street, Box 74, New York, NY 10065 USA; Department of Public Health, Supreme Council of Health, P.O. Box: 42, Doha, Qatar

**Keywords:** Seat belt, Mobile phone, Accident, Road safety, Driving, Qatar

## Abstract

**Background:**

In Qatar traffic injuries and fatalities are of serious concern. Mobile phone use whilst driving has been associated with increased risk of vehicular collisions and injuries. Seat belt use has been demonstrated to save lives and reduce the severity of road traffic injuries. Whereas previously published studies may have looked at all front passengers, this study aims to obtain reliable estimates of the prevalence of seat belt and mobile phone use among vehicle drivers in the city of Doha, Qatar. Additionally, we aim to investigate the association of these behaviors with other variables namely gender, time of the day and type of vehicle.

**Methods:**

An observational study on 2,011 vehicles was conducted in 2013. Data were collected at ten sites within Doha city over a two-week period. Two trained observers surveyed each car and recorded observations on a data collection form adapted from a form used in a 2012 Oklahoma observational study. Associations were assessed using the Chi-squared test or Fisher’s exact test. A p-value of .05 or less was considered statistically significant.

**Results:**

Overall, 1,463 (72.7 %) drivers were found using a seat belt (95 % CI: 70.8–74.7 %) and 150 (7.5 %) their mobile phones (95 % CI: 6.3–8.6 %) during the observation period. Mobile phone use was significantly associated with not using a seat belt and driving a sport utility vehicle. Significantly lower rates of seat belt use were observed in the early morning and late afternoon. No gender differences were observed.

**Discussion:**

Seatbelt use in Doha was found to be similar to countries in the region but lower than those in western countries. Also, studies from other high-income locations, reported lower rates of mobile phone use while driving than in Doha.

**Conclusions:**

Despite road traffic crashes being one of the leading causes of death in Qatar, three out of 10 drivers in Doha, Qatar, do not use a seat belt and about one in 12 use a mobile phone while driving. More efforts, in the form of awareness campaigns and increased law enforcement, are needed to improve compliance with laws requiring seat belt use and prohibiting mobile phone use while driving.

## Background

Seat belt use has been demonstrated to save lives and reduce the severity of road traffic injuries. According to the World Health Organization (WHO), the risk of death is reduced by at least 40 % among drivers and front seat passengers using a seat belt [1]. The United States National Highway Traffic Safety Administration estimated that seat belt use saved the lives of more than 12,000 vehicular passengers in the country in 2012 [2]. Mobile phone use whilst driving has been associated with an increased risk of vehicular collisions [3] and increase in reaction time to events [4]. The prevalence of seat belt and mobile phone use whilst driving in published literature has been mainly calculated from self-reported data, which, at least for seat belt use, has been shown to yield overestimates [5, 6]. Recently, observational studies have been utilized to measure such behaviors, as the methodology of such studies can lead to more accurate estimates [5].

The reliable estimation of the prevalence of seat belt and mobile phone use is crucial for public health efforts aimed at reducing the burden of injuries and fatalities from road traffic crashes. Qatar is a high-income Arab state where road traffic injuries and fatalities are of serious concern.

Qatar has witnessed rapid economic growth in the recent years with concomitant increased motorization and an increased number of mobile phone users. The number of mobile phone subscriptions increased from approximately 121,000 in 2000 to 3.3 million in 2013 [7]. Over the same period, the population increased from about 600,000 to 2.2 million [8]. Since 2010, Qatar’s traffic law mandates seat belt use for all front seat passengers and prohibits mobile phone use while driving. Heavy fines and license penalty points are imposed for those who break the law. In the case of repeat violations, the driving license may be suspended. However, the perceived enforcement of seat belt law in Qatar was rated six out of 10 (with zero being the lowest and 10 the highest) in the most recent *Global Status Report on Road Safety* [1].

Over the past years, motor vehicle crashes (MVC) were among the top five leading causes of death in Qatar, although fatality rates have reduced from 23 per 100,000 population in 2005 [9] to 14 per 100,000 population in 2010 [1]. Studies have found that in Qatar in 2001, only 8 % of those injured in MVCs were using a seat belt; this figure increased to 67 % in 2004 [10], but then decreased to 33 % in 2006–2007 [11]. All reported seat belt and mobile phone use in Qatar is based on either self-reports of drivers [12] or on hospital records of injured subjects in a MVC [10, 11]. The only exceptions were two observational studies – one undertaken on 500 vehicles at two locations near schools [13] and the other on 700 vehicles at the entrances of two universities in Qatar [14]. Since these two studies provide data from selected samples, they are not thus representative of the general population of drivers. Additional studies are warranted to obtain reliable estimates. This observational study aims to assess seat belt and mobile phone use among drivers in the city of Doha. Additionally, we aim to investigate the association of these behaviors with other variables namely gender, time of the day, and type of vehicle, and to obtain reliable estimates of these behaviors in this study population of Doha, Qatar.

## Methods

We conducted an observational study on 2,011 vehicles after receiving permission from the Traffic Department to conduct the observational study. Data were collected at 10 sites within the city of Doha during weekdays (Sunday to Thursday) over the period of two weeks in September and October 2013, between the hours of 7:00 am and 5:00 pm (excluding 12:00 pm to 3:00 pm due to extreme temperatures). The sites were chosen deliberately and included main highways and major intersections in residential, commercial and industrial areas in order to estimate vehicle driver behaviors to be representative of Doha. Two trained observers monitored each car and recorded their observations on a data collection form adapted from a form used in a 2012 Oklahoma observational study [15]. To minimize inter-observer variation, both observers reached consensus on all data fields required on the data collection form. If there was a discrepancy in any data field observation, that vehicle and driver data were excluded from the study; 40 cars and their drivers (2 % of observations) were excluded for this reason. The average duration of vehicle observation at any site was approximately thirty minutes. All observations were made on main roads for moving vehicles approaching a traffic light or roundabout, approaching a right turn prior to the traffic light intersection, or after a U-turn on traffic light. This allowed maximum visibility of driver seat belt and mobile phone use by the observers. Seat belt use was established by the observation of a proper shoulder harness by the vehicle driver. Mobile phone use was determined by observing the use of any handheld mobile phone device by the vehicle driver. Data was collected on forms documenting the time of the day, location, vehicle type, driver gender, and driver use of seat belt and/or mobile phone use. Police vehicles, buses, and ambulances were excluded. In order to estimate the prevalence of seat belt and mobile phone use the formula used for confidence interval was 1.96^2^(p)(1-p)/(Margin of Error)^2^. We assumed margin of error of 2.5 % and used p = .50 since this value maximizes the formula for adequate sample size. Using this approach we determined that a sample size of 1,540 was needed.

The Office of Research Compliance at Weill Cornell Medical College in Qatar reviewed the proposal, and determined that the current study was exempt from Qatari and United States (US) human subjects’ protection regulations and therefore did not require review by an institutional review board.

### Statistical analysis

All variables were summarized using frequency distributions. The prevalence of the two main outcomes of seat belt and mobile phone use among drivers were computed along with their 95 % confidence intervals. The association between the two main outcomes and the association between each of them with the gender of the driver, the type of vehicle, and the time of day were assessed using the Chi-squared test or Fisher’s exact test when expected cell counts fell below five. All analyses were done using STATA (version 11, Texas USA). A *p*-value of .05 or less was considered statistically significant.

## Results and discussion

Of the 2,011 vehicle drivers observed, the vast majority were male (93.7 %), and about two-thirds of the observed vehicles were either cars (37.9 %) or sports utility vehicles (SUV) (31.4 %) (Table 1).

**Table 1:** Seat belt and mobile phone use rates by gender and vehicle type

Variable	Overall	Seat belt use	*p*-value	Mobile phone use	*p*-value
Seat belt use					<.001*
Yes	1,463 (72.75 %)			52/1,463 (3.55 %)	
No	548 (27.25 %)			98/548 (17.88 %)	
Mobile phone use					
Yes	150 (7.46 %)				
No	1,861 (92.54 %)				
Gender			.491		.575
Male	1,885 (93.73 %)	1,368/1,885 (72.57 %)		139/1,885 (7.37 %)	
Female	126 (6.27 %)	95/126 (75.40 %)		11/126 (8.73 %)	
Car type			<.001*		<.001*
SUV	631 (31.38 %)	380/631 (60.22 %)^a^		86/631 (13.63 %)^a^	
Truck	161 (8.01 %)	121/161 (75.16 %)^b^		10/161 (6.21 %)^b^	
Pickup	304 (15.12 %)	234/304 (76.97 %)^b^		13/304 (4.28 %)^b,c^	
Car	762 (37.89 %)	590/762 (77.43 %)^b^		39/762 (5.12 %)^b^	
Van	112 (5.57 %)	97/112 (86.61 %)^c^		1/112 (0.89 %)^c^	
Taxi	41 (2.04 %)	41/41 (100.00 %)^d^		1/41 (2.44 %)^b,c^	
Time of day			<.001*		.261
7.00 am – 7.59 am	300 (14.92 %)	232/300 (77.33)^b,c^		15/300 (5.00 %)	
8.00 am – 9.59 am	303 (15.07 %)	219/303 (72.28)^a,c^		18/303 (5.94 %)	
10.00 am 11.59 am	605 (30.08 %)	431/605 (71.24)^a,c^		52/605 (8.60 %)	
3.00 pm – 3.59 pm	403 (20.04 %)	319/403 (79.16)^b^		32/403 (7.94 %)	
4.00 pm – 4.59 pm	400 (19.89 %)	262/400 (65.50)^a^		33/400 (8.25 %)	

*significant difference at the five percent level

^a,b,c,d^different superscript letters indicate significant differences

Overall, 1,463 (72.7 %; 95 % CI: 70.8–74.7 %) drivers were observed using a seat belt and 150 (7.5 %; 95 % CI: 6.3–8.6 %) their mobile phones. Mobile phone use was significantly lower among drivers who were wearing a seat belt as compared to drivers who were not (3.5 % versus 17.9 %, *p* < .001). There were no significant associations between gender and any of the two main outcomes. Vehicle type was significantly associated with seat belt use (*p* < .001) and with mobile phone use (*p* < .001). In particular, drivers of SUVs demonstrated lower seat belt use (60.2 %) and higher mobile phone use (13.6 %) compared to drivers of other vehicle types. Seat belt use was significantly higher in the early morning (7.00 am to 7.59 am) and early afternoon (3.00 pm to 3.59 pm) than in late morning and late afternoon (p < .001). Although mobile phone use was higher after 10 am as compared to early mornings, there was no significant increasing trend detected over the observation time periods (Table 1).

The study results show seat belt use of 73 % among vehicle drivers in Doha. This is within the two reported observational values in Doha of 43 % among university student drivers [14] and 89 % among front seat passengers near two schools [12]. The difference in university students’ prevalence could be due to the drivers’ age. University students are likely to be younger than our study population. In Qatar, self-reports of seat belt use by drivers also ranged between 45 % [16] among Qatari and non-Qatari drivers and about 80 % among Qatari drivers [12]. The prevalence of seat belt use in Doha is lower than that reported using similar direct observation methodology elsewhere such as Spain 89.5 % [17] and Michigan, US 94.7 % [18], and similar to or better than some locations such as Iran 77.9 % [19], Cuernavaca, Mexico 72.5 % [20], Kuwait 55 % [21], United Arab Emirates 29 % [22], Cairo, Egypt 16 % [20], and Lipetskaya region in Russia 55 % [23]. Although most countries have laws for mandatory seat belt use, differences in prevalence might be due to the level of enforcement of the law.

In the current study, approximately 8 % of drivers were observed using handheld mobile phone devices while driving. This point prevalence is lower than self-reported prevalences of drivers in Qatar; in one study, 73 % of drivers involved in a MVC in Qatar reported that they sometimes use a mobile phone while driving [24], whereas in another, 42 % reported using a mobile phone for calls while driving and 20 % admitted to texting while driving [12]. Studies in other high-income locations, which applied a similar methodology to this study reported lower rates of mobile phone use while driving than in Doha. For instance, Spain reported a rate of 3.8 % [17], Ireland, 2.6 % [25], and Australia, 0.6 % respectively [26]. In Iran, a similar study found the rate to be 3.6 % [27].

A significant correlation was observed between the type of vehicle driven, seat belt and mobile phone use; drivers of SUV were found to have the lowest rates of seat belt use and the highest rates of mobile phone use. This is consistent with the observational study conducted at two universities in Qatar where the prevalence of seat belt use among students driving SUVs was 34.1 %, compared to 53.4 % of students driving non-SUV vehicles [14]. Similar trends were also noted in another Qatari study using self-reports, where 67 % of small car drivers reported mobile phone use while driving, compared to 83 % of SUV drivers [24]. Similarly, SUV drivers in the United Arab Emirates (UAE) were found to be less likely to use the seat belt and more likely to commit traffic violations than non-SUV drivers [28], and similar trends were also observed in the United Kingdom [29]. In contrast, a study in the US found SUV drivers more likely to wear seat belts compared to drivers of other vehicles [30]. This difference could be due to several reasons. It is possible that SUV drivers in the US have different demographic and socioeconomic profiles than their counterparts in Qatar and the UAE. It might also be about attitudes. Some people buy larger cars as they believe them to be safer – this safety minded attitude may be why they wear a seatbelt more often in one country and also why they wear it less in another.

The significant association between seat belt and mobile phone use in the current study is in line with the other studies, where it has also been shown that the drivers who do not wear seat belts are more likely to use a mobile phone while driving [18, 29].

The significant association between seat belt use and the time of the day in the present study has also been observed in other research studies [31, 32].

Only 6 % of the drivers observed in this study were female. Only 24 % of the population of Qatar is female. Additionally, there is heavy reliance on professional vehicle drivers in the country, who are almost exclusively male. Therefore, it is possible that this number approximates the true percentage of female drivers on the road. However, additional studies are required to corroborate.

### Strengths and limitations

The observational study design makes the results of this study free from subjective bias of self-reports or data obtained from hospital records. Additionally, the use of two observers at the same site minimizes observer bias and hence improves the validity of the data. The data collected are vehicle specific, and the large sample size of the study allows high precision of the observed estimates. The diverse locations chosen allow for the possible generalizability of the study results to all of Doha.

The study is not without limitations. Our study provides point prevalence data and does not provide a complete picture of actual mobile phone use among drivers in Doha. The observed mobile phone use whilst driving could be an underestimate of the true prevalence, since we only observed vehicles for a very short period of time (approximately 10 s). Therefore, comparison of our study findings with other published studies documenting self-reported data is inappropriate. Additionally, we could not assess the effect of age or nationality on the outcome variables since it is difficult to reliably measure those when using an observational study design. However, a recent study conducted in Qatar showed no significant differences in reported seat belt use across nationalities [33]. Our observations were limited to daytime hours as the visibility is too low during nighttime to observe vehicle drivers. Our observations were also limited to weekdays. We therefore cannot comment on any differences in behavior that could have been observed at night or on weekends among vehicle drivers.

## Conclusions

Motor vehicle crashes are a leading cause of death in Qatar; despite this, three out of 10 drivers continue to neglect the use of a seat belt, and about one in 12 use a mobile phone while driving. More efforts, in the form of awareness campaigns, improved environmental conditions and increased law enforcement, are required to improve the situation. Currently, the law in Qatar does not require mandatory seatbelt use for rear passengers. The application of compulsory seat belt use for rear passengers would result in a more comprehensive coverage that can reduce motor vehicle injury [1]. Additional studies are required in order to better understand the magnitude of the problem and predictors associated with mobile phone use and or lack of seat belt use. Future research should focus on ascertaining associations, if any, between these behaviors and other demographic variables such as the driver’s age, nationality, marital status, and education, as well as other relevant parameters such as time and day of observation, particularly at nighttime and weekends.

## Abbreviations

MVC: Motor vehicle crashes
SUV: Sports utility vehicle
UAE: United Arab Emirates
US: United States (of America)
WHO: World Health Organization

## References

[1] Global status report on road safety 2013: supporting a decade of action: summary. Geneva: World Health Organization 2013. http://www.who.int/violence_injury_prevention/road_safety_status/2013/en/. Accessed June 2015

[2] Traffic Safety Facts: 2012 Data. Washington, DC: NHTSA’s National Center for Statistics and Analysis 2014 Contract No. DOT HS 812 016. http://www-nrd.nhtsa.dot.gov/Pubs/812016.pdf. Accessed June 2015.

[3] McEvoy SP, Stevenson MR, McCartt AT, Woodward M, Haworth C, Palamara P (2005). Role of mobile phones in motor vehicle crashes resulting in hospital attendance: a case-crossover study. BMJ.

[4] Caird JK, Willness CR, Steel P, Scialfa C (2008). A meta-analysis of the effects of cell phones on driver performance. Accid Anal Prev.

[5] Özkan T, Puvanachandra P, Lajunen T, Hoe C, Hyder A (2012). The validity of self-reported seatbelt use in a country where levels of use are low. Accid Anal Prev.

[6] Parada MA, Cohn LD, Gonzalez E, Byrd T, Cortes M (2001). The validity of self-reported seatbelt use: Hispanic and non-Hispanic drivers in El Paso. Accid Anal Prev.

[7] Statistics: Time series by country: Mobile-Cellular Subscriptions [database on the Internet]. International Telecommunication Union. 2014. Available from: http://www.itu.int/en/ITU-D/Statistics/Documents/statistics/2014/Mobile_cellular_2000-2013.xls. Accessed: 15 feb 2015.

[8] Qatar Data [database on the Internet]. The World Bank Group. Available from: http://data.worldbank.org/country/qatar. Accessed: 18 Aug 2015.

[9] Bener A (2005). The neglected epidemic: road traffic accidents in a developing country, State of Qatar. Int J Inj Contr Saf Promot.

[10] Bener A, Al Humoud SMQ, Price P, Azhar A, Khalid MK, Rysavy M (2007). The effect of seatbelt legislation on hospital admissions with road traffic injuries in an oil-rich, fast-developing country. Int J Inj Contr Saf Promot.

[11] Munk M-D, Carboneau DM, Hardan M, Ali FM (2008). Seatbelt use in Qatar in association with severe injuries and death in the prehospital setting. Prehosp Disaster Med.

[12] Burgut HR, Bener A, Sidahmed H, Albuz R, Sanya R, Khan WA (2010). Risk factors contributing to road traffic crashes in a fast-developing country: the neglected health problem. Ulus Travma Acil Cerrahi Derg.

[13] Darabi Golshani AM, Nikraz H, Darabi Golshani Z (2009). Observational survey of seat belt usage in Doha, Qatar.

[14] Shaaban K (2012). On road observational survey of seat belt Use among young drivers in Qatar. Proceedings of measuring behavior 2012, 8 th international conference on methods and techniques in behavioral research.

[15] James TE, Hall K (2010). Seat belt observation study summer 2010.

[16] Bener A, Ã–zkan TR, Lajunen T (2008). The driver behaviour questionnaire in arab gulf countries: Qatar and United Arab Emirates. Accid Anal Prev.

[17] Martínez-Sánchez JM, Curto A, Fu M, Martínez C, Sureda X, Ballbè M (2014). Safety belt and mobile phone usage in vehicles in Barcelona (Spain). Gac Sanit.

[18] Russo BJ, Kay JJ, Savolainen PT, Gates TJ (2014). Assessing characteristics related to the use of seatbelts and cell phones by drivers: application of a bivariate probit model. J Safety Res.

[19] Sadeghnejad F, Niknami S, Hydarnia A, Montazeri A (2014). Seat-belt use among drivers and front passengers: an observational study from the Islamic Republic of Iran. East Mediterr Health J.

[20] Vecino-Ortiz AI, Bishai D, Chandran A, Bhalla K, Bachani AM, Gupta S (2014). Seatbelt wearing rates in middle income countries: a cross-country analysis. Accid Anal Prev.

[21] Al-Saleh OI, Koushki PA (2007). Assessment of urban traffic infractions in Kuwait. Transportation Research Record. J Transport Res Board.

[22] Barss P, Al-Obthani M, Al-Hammadi A, Al-Shamsi H, El-Sadig M, Grivna M (2008). Prevalence and issues in non-use of safety belts and child restraints in a high-income developing country: lessons for the future. Traffic Inj Prev.

[23] Ma S, Tran N, Klyavin VE, Zambon F, Hatcher KW, Hyder AA (2012). Seat belt and child seat use in Lipetskaya Oblast, Russia: frequencies, attitudes, and perceptions. Traffic Inj Prev.

[24] Bener A, Crundall D, Özkan T, Lajunen T (2010). Mobile phone use while driving: a major public health problem in an Arabian society, State of Qatar—mobile phone use and the risk of motor vehicle crashes. J Public Health.

[25] Gilroy I, Donnelly N, Matthews W, Doherty K, Conlon G, Clarke AT (2013). Smoking in vehicles is lower than mobile telephone use while driving, but is socially patterned. Ir Med J.

[26] Wundersitz LN (2014). Phone use while driving: results from an observational survey. Traffic Inj Prev.

[27] Ashrafi Asgarabad A, Naghibzadeh Tahami A, Khanjani N (2012). Exposure to hand-held mobile phone use while driving among Iranian passenger car drivers: an observational study. J Inj Violence Res.

[28] Bener A, Al Maadid MGA, Özkan T, Al-Bast DAE, Diyab KN, Lajunen T (2008). The impact of four-wheel drive on risky driver behaviours and road traffic accidents. Transp Res Part F Traffic Psychol Behav.

[29] Walker L, Williams J, Jamrozik K (2006). Unsafe driving behaviour and four wheel drive vehicles: observational study. BMJ.

[30] Eluru N, Bhat CR (2007). A joint econometric analysis of seat belt use and crash-related injury severity. Accid Anal Prev.

[31] Masten SV (2007). Do states upgrading to primary enforcement of safety belt laws experience increased daytime and nighttime belt use?. Accid Anal Prev.

[32] Chaudhary NK, Tison J, Casanova T (2010). The effects of Maine’s change to primary seat belt law on seat belt use and public perception and awareness. Traffic Inj Prev.

[33] Bener A, Verjee M, Dafeeah EE, Yousafzai MT, Mari S, Hassib A (2013). A Cross “Ethnical” Comparison of the Driver Behaviour Questionnaire (DBQ) in an Economically Fast Developing Country. Glob J Health Sci.

